# Medicinal plants popularly used in the Xingó region – a semi-arid location in Northeastern Brazil

**DOI:** 10.1186/1746-4269-2-15

**Published:** 2006-03-23

**Authors:** Cecília de Fátima CBR Almeida, Elba Lúcia Cavalcanti de Amorim, Ulysses Paulino de Albuquerque, Maria Bernadete S Maia

**Affiliations:** 1 Departamento de Biologia, Área Botânica, Laboratório de Etnobotânica Aplicada, Universidade Federal Rural de Pernambuco, Rua Dom Manoel de Medeiros s/n, Dois Irmãos, Recife, Pernambuco, 52171–030, Brazil; 2 Departamento de Ciências Farmacêuticas, Universidade Federal de Pernambuco, Cidade Universitária, Recife, Pernambuco, Brazil; 3 Departamento de Fisiologia, Universidade Federal de Pernambuco, Cidade Universitária, Recife, Pernambuco, Brazil

## Abstract

The aim of this study was to identify plant species among the diverse flora of the *caatinga *ecosystem that are used therapeutically. Research was undertaken in the municipalities of Piranhas and Delmiro Gouveia, in the Xingó region (state of Alagoas, NE Brazil). In order to identify the medicinal plants used in this region, semi-structured questionnaires were applied. The species cited were collected and sent to the Xingó Herbarium for taxonomic analysis. The relative importance (RI) of each species cited was calculated to verify their cultural importance. The therapeutic indications attributed to the species were classified under 16 body systems. A total of 187 medicinal species were cited, from 64 families and 128 genera. The main indications for medicinal plant use were against common colds, bronchitis, cardiovascular problems, kidney problems, inflammations in general, and as tranquilizers. Approximately 16% (30 plant species) were versatile in relation to their use, with an Relative Importance value over 1, having been indicated for up to nine body systems. The body systems that stood out the most were: the respiratory system, the gastrointestinal system, and infectious diseases. Most cited plant parts used for medicinal purposes were flowers, leaves, and inner stem bark.

## Introduction

Medicinal plants constitute the base of heath care systems in many societies. The recovery of the knowledge and practices associated with these plant resources are part of an important strategy linked to the conservation of biodiversity, the discovery of new medicines, and the bettering of the quality of life of poor rural communities. Ethnobotanical studies of medicinal plants have taken many paths, sometimes testing hypotheses of use and knowledge [1-6] or sometimes describing the use of plants in given cultural contexts [7].

Only limited research had been done in Brazil on the use of natural medicines, although in the last 15 years more work has been initiated on the use of medicinal plants by communities living in diverse ecosystems [8-17]. The caatinga and the cerrado, for example, are two poorly studied biomes from an ethnobiological perspective. In this sense, the caatinga is a unique biome in the world in terms of the extreme heterogeneity of its vegetation physiognomy and floristic composition [18]. As such, the present work seeks to contribute to our knowledge of the medicinal plants used by the rural inhabitants of the caatinga region.

*Caatinga *vegetation is generally composed of spiny species, most of them caducifolious, which lose their leaves at the beginning of the dry season, as well as succulent or leafless plants, bromeliads and cacti [19-22]. Due to its mosaic-like pattern, with several characteristic and endemic species associated with specific soil, climate, and landscape conditions, the *caatinga *is extremely susceptible to biodiversity loss [18]. However, only 1.4% of this ecosystem is protected in official Conservation Areas, and some of *caatinga *vegetation types are located outside of these areas [18].

Despite the generally adverse environmental conditions, the *caatinga *is occupied by an extractivist society [23] heavily using the native vegetation, especially to supply energetic needs [24], as well as raising sheep and goats (free ranging) and maintaining small bean, rice, corn, and manioc plantations. Demographic growth brings structural problems to the food production system, especially when combined with the region's extremely dry climate. Soil deterioration, decreasing in biodiversity, and desertification [23] all bring misery to the local populations [23], and malnutrition and health problems are very common.

Studies on the knowledge and use of natural resources by local populations may contribute to finding economic alternatives for these populations, especially in terms of the use of natural resources for treating health problems. Despite being widely known and used, the harvesting of medicinal plants by local populations may have a low impact on native vegetation depending on the demand and the kind of product extracted [25,16,27]. In this paper we will present the results of a descriptive study of the medicinal plants of the *caatinga *ecosystem in the Xingó region, in order to identify the plant species used therapeutically and provide baseline information for future pharmacological and phytochemical studies.

## Methods

### Study area

**Figure 1:**
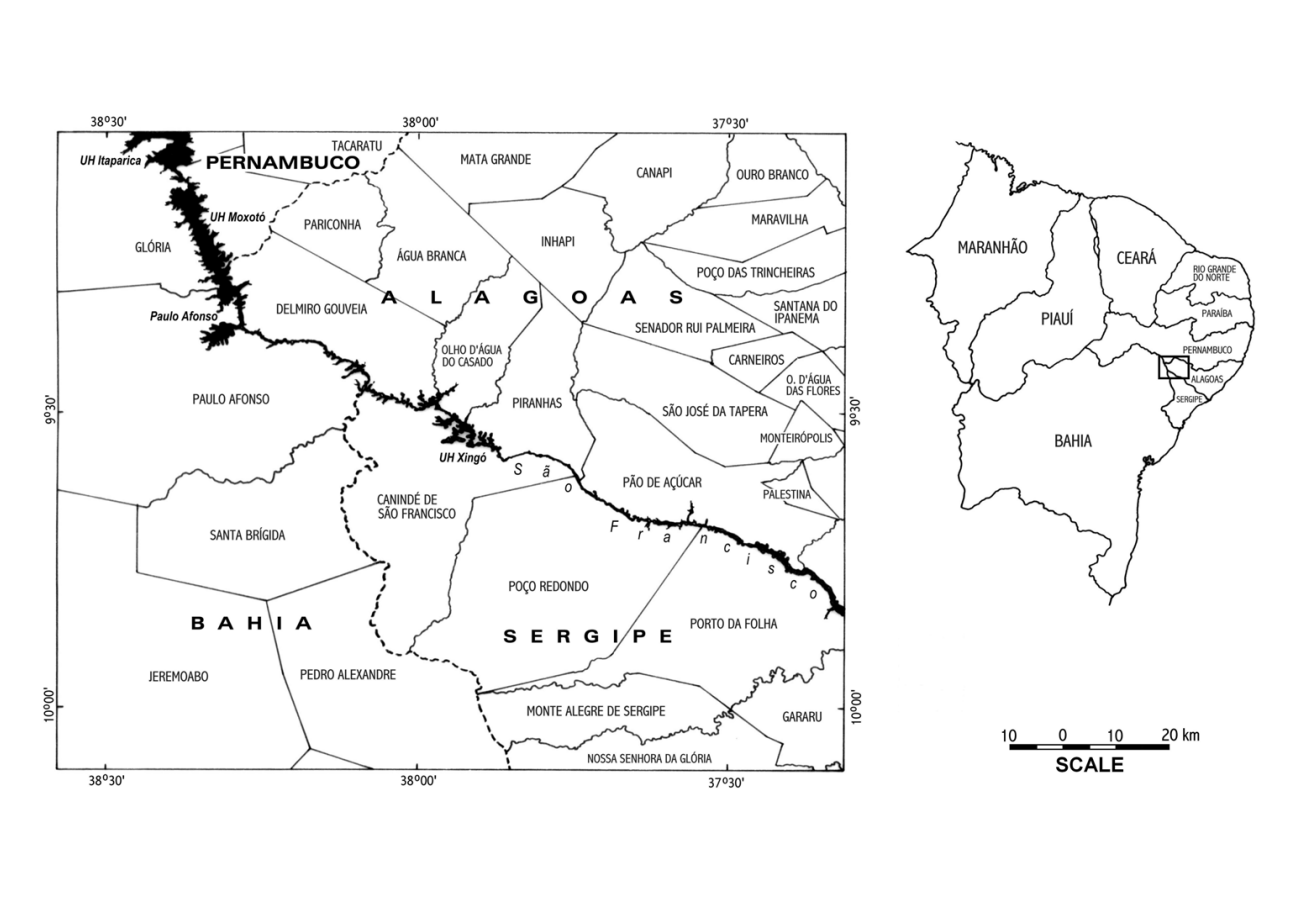
Location of the ethnobotanical and botanical data collections of medicinal plants cited by the population in two municipalities of the Xingó region (Northeastern Brazil).

The study was undertaken in a 7845 km^2 ^area in two municipalities near the Xingó Hydroelectric Power Plant, in the São Francisco Valley, bordering the states of Bahia, Alagoas, and Sergipe (central point: 09° 36' 96" south latitude and 36° 50' 88" west longitude) [28] (Figure 1). During the 1990s, a multidisciplinary initiative named the Xingo' Program was implemented in this region by the National Council of Scientific and Technologic Development (Conselho Nacional de Desenvolvimento Científico e Tecnológico–CNPq), in conjunction with the São Francisco Hydroelectric Company (Companhia Hidro Elétrica do São Francisco–CHESF). The São Francisco River runs through the central portion of this region in a northwest/southeast direction. The climate is semi-arid and hot, with scarce rainfall and long periods of drought. Annual precipitation averages 600–750 mm [29].

Interviews were held in the municipalities of Piranhas (9°37' S, 37°45' W) and Delmiro Gouveia (9°38' S, 37°99' W), state of Alagoas (Figure 1). Piranhas occupies 407 Km^2^, and is located in the state of Alagoas, in the Alagoas *sertão *mesoregion and the São Francisco *sertão *microregion. The total population of the municipality is 20007, of which 1340 live in the urban zone and 18667 in the rural zone; only 9068 of these inhabitants are literate [30]. Delmiro Gouveia occupies 607 Km^2^, and is located in the state of Alagoas, in the Alagoas *sertão *mesoregion and the São Francisco *sertão *microregion. The total population of the municipality is 42995, of which 33563 live in the urban zone and 9432 in the rural zone; 23828 of these inhabitants are literate [30].

### Data collection

Information was obtained from 339 interviews (282 with inhabitants of Piranhas and 57 in Delmiro Gouveia), carried out between 1999 and 2000, using a standard form (Table 1). The interviews were made by 26 Health Agents, chosen because of their direct access to the local inhabitants. Health agents are employed by the municipal governments to provide primary health care in the local households. These workers were given short courses about medicinal plants and usage of questionnaires in order to avoid problems related to research techniques. The health workers visited all the houses on their normal work route, and interviewed the oldest household member. Interviews varied in duration according to the degree of knowledge of medicinal plants of each interviewee. The informants were questioned about the medicinal plants they use, their mode of usage, and the parts used; information was also gathered on access to the plants and restrictions on their use, as well as data concerning the social-economic profile of the persons interviewed, such as their age, sex, schooling, and profession

**Table 1:** Basic model of the interview form

Name of the person interviewed:		
Date of birth: ____/____/______	Sex: F () M ()	
Address:		
Schooling:	Profession:	
Do you use medicinal plants?	Which ones?	
What types of diseases can be treated with these plants?		
Which plant parts are used?		
() Roots	() Stem	() Flowers
() Leaves	() Bark	() Seeds
() Fruits	() Entire plant	
How is the medicine prepared?		
() Decocted	() Tincture	() Syrup
() Infusion	() Maceration	() Other
Municipality:	Date:	
Name of the Health Agent:		

Botanical collections were undertaken in different localities within the municipalities of Piranhas and Delmiro Gouveia (where the interviews were held), with the aid of local field assistants who had completed ethnobotanical information courses. Due to the drought in the region at the time, which made plant collections difficult, collections were also made in the municipalities of Olho d'Água do Casado in the state of Alagoas, and in Canindé do São Francisco and Poço Redondo in the state of Sergipe. Representative voucher specimens of the studied material were deposited in the Xingó Herbarium, in the city of Canindé do São Francisco (state of Sergipe), and others in the IPA Herbarium (Empresa Pernambucana de Pesquisa Agropecuária), Recife (Pernambuco). The collection of botanical material was performed under the direction of the biologist Débora Coelho.

### Data analysis

Initially, the information about the popular uses of the species collected, along with botanical information, was compiled into a database. The species were listed in alphabetical order by family, popular name in the region, medicinal use, part used, and herbarium number.

The relative importance (RI) of the species cited was calculated according to [31]. Relative importance was calculated according to the following formula, with "2", being the highest possible value, indicating the most versatile species. The most versatile species are those that have the greatest number of medicinal properties: RI = NCS + NP, where NCS (Number of Body systems) is the number of body systems treated by a given species (NCSS) divided by the total number of body systems treated by the most versatile species (NSCSV). The number of properties (NP) is obtained by the relationship between the number of properties attributed to a species (NPS) divided by the total number of properties attributed to the most versatile species (NPSV). For example: to a given species ***x***, the following attributes (properties) were indicated: pneumonia, bronchitis, and asthma – all within the category (body system) "problems of the respiratory system"; while species ***y ***was considered more versatile as it had a larger number of cited properties (bronchitis, asthma, ameba parasites, headache, blood thinning, fever), and 5 body systems. As such, the RI of species ***x ***would be: (NCS = 1/5) + (NP = 3/6) = 0.7.

In using this technique, we are assuming that a plant is more important when it is most versatile [32]. This analysis has some limitations, however, for a plant with few uses, but which is used frequently by many people, would tend to be awarded only low values. Nonetheless, this quantitative technique, as are many others found in the ethnobotanical literature [32], are measures of folk knowledge and interpretations of their use must be carefully made.

Popular indications were distributed among the categories [33]: infectious diseases (ID); parasite-related diseases (PRD); diseases of the endocrine glands, nutrition, and metabolism (DENM); diseases of the blood and hematopoietic organs (DB); diseases of the skeletal, muscle, and connective tissues (DSMO); diseases of the skin and subcutaneous cellular tissues (DSSC); problems of the visual system (PVS); problems of the nervous system (PNS); problems of the circulatory system (PCS); problems of the respiratory system (PRS); problems of the digestive system (PDS); problems of the genitourinary system (PGUS); sexual impotence (SI); undefined pains and illnesses (UPI); sexually transmitted diseases (STD); neoplasie (NP). Careful attention was paid to the accuracy of the information gathered, and included interviews with local informants. However, the information concerning local uses (Table 1) is essentially a direct transcription of information gathered during the interviews. This data was used in the calculations that follow, and obey the same principals as the calculations of Relative Importance.

A technique by Trotter and Logan [34] was used to identify which categories were most important in the interviews; it is based on the "informants' consensus" (ICF) and shows which groups of plants require more in-depth studies. The maximum ICF value possible is 1, when there is total consensus among the informants about the medicinal plants for a given category. To this end, the indications were grouped in the categories described above for a more objective analysis, where the ICF formula is:

ICF = nar-na/nar-1, where:

nar = sum of the uses registered by each informant in a given category;

na = number of species indicated in that category.

## Results and discussion

Of the 339 interviewees, 92% were women and only 8% were men; 26% were aged 31–40 years, 20% were between 41–50, and 19.4% were in the 21–30 age range. Women were predominantly represented in the sampling due to the fact that working men were rarely found at home during our visits. In terms of the formal schooling of the informants, 63% were still in school or had finished elementary school; 17% were illiterate; and only 0.9% finished or were still in college.

In relation to use-preference, 60.7% of the interviewees preferred buying at pharmacies that sell industrialized phytotherapeutic products (but that do not correspond to plants available in the local region). Some interviewees commented: "I buy in the herbal pharmacies because it's cheaper". It is also interesting to note what a 53-year old male interviewee said: "Medicinal plants, they are my pharmacy". Still another interviewee, a 29-year old female, explained: "When I had no knowledge about the effects they could cause on me, I bought both, and now that I know that medications taken without a medical prescription are bad for your health, so I only take medications that are prescribed by a doctor and medicinal plants when I already know their effects".

A total of 187 medicinal species from 64 families and 128 genera were documented (see Additional file 1), an expressive number when compared to other surveys undertaken in the *caatinga*, in which the number of species varied from 48 to 97 [25,35]. Of the species recorded here, approximately 50% were native, while the other half was composed of exotic species (species native to other ecosystems or other parts of the world).

About 16% of the species (30 spp.) had high use-versatility (RI >1) and were indicated for up to nine infirmity categories (see Additional file 1): *Senna splendida *(Vogel.) H. S. Irwin & Barneby (1.875), *Lantana *sp. (1.857), *Capparis jacobinae *Moric. (1.589), *Lippia *sp. (1.428), *Skytanthus hancorniaefolius *Miers. (1.357), *Caesalpinia ferrea *Mart. ex Tul., *Bauhinia cheilantha *(Bong.) Stend., *Acacia bahiensis *Benth., and *Chamaesyce hyssopifolia *Small (1.339). Only one of these species was herbaceous; the remaining were trees or bushes. This may be related to the fact that in the *caatinga *trees are a resource that is available all year long, unlike herbs, which are limited by the scarce rainfall [25].

Among the species demonstrating the highest relative importance values in this study, *Senna occidentalis *Link. has scientifically proven antimicrobial activity against fungi and bacteria, and *Bauhinia cheilantha *(Bong.) Stend. has been shown to reduce cholesterol and triglyceride levels [36]. There is little scientific information available concerning *B. cheilantha*, and most publications concern species of the same genus that are widely used in Brazilian folk medicine, such as *Bauhinia forficata *Link. Recent studies with *S. occidentalis *indicate its effectiveness as a laxative, though it may cause intestinal disturbances with prolonged use [37].

Some authors consider that resource availability and the real needs of a given population influence traditional botanical knowledge [38]. Nevertheless, the list of plants described above does not match that which is normally reported for the *caatinga *medicinal flora, where the most important species are usually: *Anacardium occidentale *L.*, Sideroxylon obtusifolium *(Roem and Schult.) T. D. Penn., and *Myracrodruon urundeuva *(Engl.) Fr. All. [25,33,39]. These three species stand out for their versatility and for being most frequently cited.

For the population studied, the infirmity categories with the greatest consensus among informants were: problems of the respiratory system, problems of the digestive system, and infectious diseases. All of these have high cultural importance in the region (Table 2). This result matches that seen in other communities of the semi-arid region of NE Brazilian [25,40], in which the largest number of species are cited for the treatment of respiratory problems, inflammations in general, and intestinal problems. This indicates a high incidence of these types of diseases in the region, possibly due to the precarious socioeconomic and sanitary conditions of these populations.

**Table 2:** Informant consensus for diseases treated with medicinal plants used by the population in two municipalities of the Xingó region (Northeastern Brazil)

**Categories**	ICF*	**Number of uses mentioned**	**Number of plant species**
Problems of the respiratory system	0.350	64	42
Problems of the digestive system	0.210	71	56
Infectious diseases	0.160	45	38
Diseases of the skin and subcutaneous cellular tissues	0.125	9	8
Problems of the circulatory system	0.120	51	45
Problems of the genitourinary system	0.100	66	60
Problems of the nervous system	0.070	28	26
Undefined pains and illnesses	0.030	35	34
Diseases of the blood and hematopoietic organs	0.000	20	20
Diseases of the endocrine glands, nutrition, and metabolism	0.000	13	13
Diseases of the skeletal, muscle, and connective tissues	0.000	13	13
Parasite-related diseases	0.000	8	8
Neoplasies	0.000	7	7
Sexual impotence	0.000	5	5
Sexually transmitted diseases	0.000	5	5
Problems of the visual system	0.000	2	2

* ICF: Informant consensus factor

Lack of consensus among informants may be due to their diverse backgrounds or to their different sources of information, but most likely reflects the fact that the study area was very large and local knowledge concerning these species may vary greatly between these sites. In a study undertaken in the state of Pernambuco, Almeida and Albuquerque [33] used principal components analysis to analyze a group of 20 interviewees, finding that three of them differed greatly from the rest, as one gave a very high number of citations, the consensus of a second was very different from that of the rest, and a third probably diverged due to the place of birth.

The most cited medicinally used plant parts were flowers (35%), leaves (33%), and inner stem bark (10%). It is curious that flowers were the most cited plant part; usually the stem or its bark is preferred for medicinal use in the *caatinga *due to its continuous temporal availability [25,41,42]. However, it is important to note that flowers were cited both alone and accompanied by other plant parts, such as the leaf or inner stem bark. This may indicate that when a certain plant part is not available, the population collects another part of the same plant or an entirely different plant for the same purpose.

The biological activity of most of the species cited has not yet been studied, indicating the need of more studies with *caatinga *plants. There are apparently no published works focusing on the activities commonly attributed to the species cited in our survey. Curiously, these plants were not important in other ethnobotanical surveys undertaken in semi-arid regions, while other less-valued species in the current survey (such as *Myracrodruon urundeuva, Schinopsis brasiliensis*, and *Anadenanthera colubrina*) are highly valued in other communities [25,33,42].

Considering that the *caatinga *has a high diversity of medicinal plants that are still poorly studied, more phytochemical and pharmacological studies are necessary in order to test popular indications and search for new pharmaceuticals. Additional studies are also necessary to identify possible links between a plant's chemical composition and its habit and life strategy, and to determine how human populations in the *caatinga *select and use these plants.

## Additional material

Medicinal plants cited by the population in two municipalities of the Xingó region (Northeastern Brazil). RI = Relative importance. *All plants are preferentially used fresh.

## Supplementary Material

Additional File 1 Medicinal plants cited by the population in two municipalities of the Xingó region (Northeastern Brazil). RI = Relative importance. *All plants are preferentially used fresh. Click here for file

## References

[B1] Vandebroek I, Calewaert J, De jonckheere S, Sanca S, Semo L, Van Damme P, Van Puyvelde L, De Kimpe N (2004). Use of medicinal plants and pharmaceuticals by indigenous communities in the Bolivian Andes and Amazon. Bulletin of the World Health Organization.

[B2] Monteiro JM, Albuquerque UP, Lins-Neto EMF, Araújo EL, Amorim ELC (2006). Use patterns and knowledge of medicinal species among two rural communities in Brazil's semi-arid northeastern region. J Ethnopharmacol.

[B3] Stepp JR, Moerman DE (2001). The importance of weeds in ethnopharmacology. Journal of Ethnopharmacology.

[B4] Stepp JR (2004). The role of weeds as sources of pharmaceuticals. Journal of Ethnopharmacology.

[B5] Voeks RA (1996). Tropical Forest healers and habitat preference. Economic Botany.

[B6] Reyes-Garcia V, Vadez V, Huanca T, Leonard W, Wilkie D (2005). Knowledge and consumption of wild plants: a comparative study in two Tsimane' villages in the Bolivian Amazon. Ethnobotany Research & Applications.

[B7] Gazzaneo LRS, Lucena RFP, Albuquerque UP (2005). Knowledge and use of medicinal plants by local specialists in a region of Atlantic Forest in the state of Pernambuco (Northeast Brazil). Journal of Ethnobiology and Ethnomedicine.

[B8] Albuquerque UP (2001). The use of medicinal plants by the cultural descendants of african people in Brazil. Acta Farmacéutica Bonaerense.

[B9] Monteiro JM, Almeida CFCBR, Albuquerque UP, Lucena RFP, Florentino ATN, Oliveira RLC (2006). Use and traditional management of *Anadenanthera colubrina *(Vell.) Brenan in the semi-arid region of northeastern Brazil. Journal of Ethnobiology and Ethnomedicine.

[B10] Rodrigues E, Carlini EA (2005). Ritual use of plants with possible action on the central nervous system by the Krahô Indians, Brazil. Phytotherapy Research.

[B11] Rodrigues E, Carlini EA (2004). Plants used by a Quilombola group in Brazil with potential central nervous system effects. Phytotherapy Research.

[B12] Hanazaki N, Tamashiro JY, Leitão-Filho HF, Begossi A (2000). Diversity of plant uses in two Caiçaras communities from the Atlantic Forest coast, Brazil. Biodiversity and Conservation.

[B13] Rossato SC, Leitão-Filho H, Begossi A (1999). Ethnobotany of caiçaras of the Atlantic Forest Coast (Brasil). Economic Botany.

[B14] Di Stasi LC, Oliveira GP, Carvalhaes MA, Queiroz-Junior M, Tien OS, Kakinami SH, Reis MS (2002). Medicinal plants popularly used in the Brazilian Tropical Atlantic Forest. Fitoterapia.

[B15] Figueiredo GM, Leitão-Filho HF, Begossi A (1993). Ethnobotany of Atlantic Forest coastal communities: diversity of plant uses in Gamboa (Itacuruçá Island, Brazil). Human Ecology.

[B16] Figueiredo GM, Leitão-Filho HF, Begossi A (1997). Ethnobotany of Atlantic Forest Coastal Communities: II. Diversity of plant uses at Sepetiba Bay (SE Brasil). Human Ecology.

[B17] Hanazaki N, Leitão-Filho HF, Begossi A (1996). Uso de recursos na Mata Atlântica: o caso da Ponta do Almada (Ubatuda, Brasil). Interciencia.

[B18] Sampaio EVSB, Souto A, Rodal MJN, Castro AAJF, Hazin C, Fundação Grupo Esquel Brasil (1994). Caatingas e cerrados do NE – biodiversidade e ação antrópica. Conferência Nacional e Seminário Latino-americano da desertificação: Ceará.

[B19] Andrade-Lima D (1981). The caatinga dominium. Revista Brasileira de Botânica.

[B20] Andrade-Lima D, Prance GT (1982). Present-day Forest refuges in northeastern Brazil. Biological diversification in the tropics.

[B21] Veloso HP, Góes-Filho L (1982). Fitogeografia brasileira: classificação fisionômica-ecológica da vegetação neotropical. Boletim Técnico do Projeto RADAMBRASIL.

[B22] Souza MJN, Martins MLR, Soares ZML, Freitas-Filho MR, Almeida MAG, Pinheiro FSA, Sampaio MAB, Carvalho GMBS, Soares AML, Gomes ECB, Silva RA (1994). Redimensionamento da região semi-árida do Nordeste do Brasil. Conferência Nacional e Seminário Latino-americano da desertificação: 1994; Ceará.

[B23] Drumond MA, Kill LHP, Lima PC (2002). Estratégias para o uso sustentável da biodiversidade da caatinga Seminário: Biodiversidade da Caatinga.

[B24] Rodal MJN, Sampaio EVSB (2002). A vegetação do bioma caatinga. Vegetação & Flora da Caatinga: 2002; Recife Edited by Sampaio EVSB, Giulietti AM, Virginio J, Gamarra-Rojas CFL.

[B25] Albuquerque UP, Andrade LHC (2002). Uso de recursos vegetais da caatinga: o caso do Agreste do estado de Pernambuco (Nordeste do Brasil). Interciencia.

[B26] Silva MA, EMBRAPA-DDT (1986). Plantas Úteis da Caatinga. Anais do Simpósio sobre caatinga esua exploração racional: 1986; Brasília.

[B27] Sampaio EVSB, Sampaio EVSB, Giulietti AM, Virginio J, Gamarra-Rojas CFL (2002). Uso das plantas da caatinga. Vegetação & Flora da Caatinga: 2002; Recife.

[B28] RADAMBRASIL, RADAMBRASIL (1983). Vegetação. Geologia, geomorfologia, pedologia, vegetação, uso potencial da terra. Levantamento de Recursos Vegetais 30: 1983; Rio de Janeiro.

[B29] Assis JS (1999). Centro regional de estudo sobre a caatinga: zoneamento ambiental e plano de unidades de conservação da caatinga no estado de Alagoas (escala 1: 100x000).

[B30] Instituto Brasileiro de Geografia e Estatística (IBGE) (2000). Malha municipal digital do Brasil Rio de Janeiro.

[B31] Bennett BC, Prance GT (2000). Introduced plants in the indigenous pharmacopoeia of Northern South America. Economic Botany.

[B32] Silva VA, Albuquerque UP, Albuquerque UP, Lucena RFP (2004). Técnicas para análise de dados etnobotânicos. Métodos e técnicas na pesquisa etnobotânica: 2004; Recife.

[B33] Almeida CFCBR, Albuquerque UP (2002). Uso e conservação de plantas e animais medicinais no estado de Pernambuco (Nordeste do Brasil): um estudo de caso. Interciencia.

[B34] Trotter R, Logan M (1986). Informant consensus: new approach for identifying potentially effective medicinal plants. Indigenous Medicine and Diet: Biobehavioural Approaches.

[B35] Costa-Neto EM, Oliveira MVM (2000). The use of medicinal plants in county of Tanquinho, state of Bahia, northeastern Brazil. Brazilian Journal of Medicinal Plants.

[B36] Lorenzi H, Matos FJA (2002). Plantas medicinais no Brasil – Nativas e Exóticas.

[B37] Nadal SR, Calore EE, Monzione CR, Puga FR, Perez NM (2003). Effects of long-term administration of *Senna occidentalis *seeds in the large bowel of rats. Pathology Research and Practice.

[B38] Albuquerque UP (2005). Introdução à etnobotânica.

[B39] Almeida CFCBR, Silva TCL, Amorim ELC, Maia MBS, Albuquerque UP (2005). Life strategy and chemical composition as predictors of the selection of medicinal plants from the Caatinga (Northeast Brazil). Journal of Arid Environments.

[B40] Silva VA (1997). Etnobotânica dos índios Xucuru com ênfase às espêcies do Brejo da Serra do Orobó (Pesqueira-PE).

[B41] Albuquerque UP, Silva ACO, Andrade LHC (2005). Use of plant resources in a seasonal dry forest (northeastern Brazil). Acta Botanica Brasílica.

[B42] Silva ACO, Albuquerque UP (2005). Woody medicinal plants of the caatinga in the state of Pernambuco (northeast Brazil). Acta Botanica Brasílica.

