# Supplementary material for: Medicinal plants popularly used in the Xingó region – a semi-arid location in Northeastern Brazil
Source: J Ethnobiol Ethnomed. 2006 Mar 23;2:15. doi: 10.1186/1746-4269-2-15 (PMC1444943; doi:10.1186/1746-4269-2-15)
Supplement: Additional File 1 — Medicinal plants cited by the population in two municipalities of the Xingó region (Northeastern Brazil). RI = Relative importance. *All plants are preferentially used fresh. [file 1746-4269-2-15-S1.pdf]

Additional Material. Medicinal plants cited by the population in two municipalities of the Xingó region (Northeastern Brazil). RI = Relative importance. *All plants are preferentially used fresh.

**FAMILY/ SCIENTIFIC NAME**<br>**Common name**	**USES FORMS/ INDICATIONS**	**PLANT COMPONENT***	**RI**	**HERBARIUM NUMBER**
ACANTHACEAE				
Ruellia asperula (Ness) Lindau<br>Candeia or camará-candeia	Macerated: Bronchitis, asthma, flu, fever, uterus inflammation	Leaf, flower, root	1.196	396, 397, 398
Ruellia sp.	Macerated: Expel catarrh	Leaf, flower	0.267	395, 400
AMARANTHACEAE				
Alternanthera brasiliana (L.) Kuntze<br>Terramicina	Decoction: Numerous types of inflammations, worms	Leaf, flower	0.535	403, 404
Alternanthera sp.1<br>Erva-branca	Decoction: Menstrual cramps	Leaf, flower	0.267	402
Alternanthera sp.2	Decoction: Urinate keep, kidneys stone, sedative	Leaf, flower	0.678	405, 406
AMARYLIDACEAE				
Amarylis beladona L.<br>Cebola-braba	Syrup: Pneumonia, bronchitis	Bulb	0.410	407
ANACARDIACEAE				
Myracrodruon urundeuva Fr. All.<br>Aroeira	Decoction/Bath: Numerous types of inflammations	Inner bark	0.267	411
Schinopsis brasiliensis Engl.<br>Braúna	Decoction/Bath: Numerous types of inflammations, sexual impotence	Inner bark, leaf	0.535	409, 410
Spondias tuberosa Arr. Cam.<br>Umbuzeiro	Decoction: Diabetes	Inner bark	0.267	408
APOCYNACEAE				
Allamanda blanchetti A. DC.<br>Sete-patacas-roxas or jasminho	Infussion: Heart (Cardiac problems), high blood pressure	Leaf, flower	0.410	419, 420
Aspidosperma pyrifolium Mart.<br>Pereiro	Infusion: Diarrhea, sedative, heart (Cardiac problems)	Inner bark, flower	0.803	421
Mandevilla tenuifolia Lindl.<br>Flor-de-Sto. Antonio	Decoction: Heart (Cardiac problems)	Leaf, flower	0.267	412

Skytanthus hancorniaefolius Miers.	Decoction: Sedative, insomnia, high blood pressure, heart (Cardiac problems), asthma, flu	Inner bark, leaf, flower	1.357	413, 416, 417
ARACEAE				
Anthurium affine Schott.<br>Folha-larga or palmeirão-brabo	Decoction: Diabetes, heart (Cardiac problems), flu, blood make fine	Leaf, flower	1.071	422, 424, 425, 426, 427
Dracontium sp.<br>Milho-de-macaco	Decoction or macerated: Rheumatism	Leaf, root	0.267	423
ARISTOLOCHIACEAE				
Aristolochia brasiliensis Mart. ex Zucc.<br>Jarrinha	Decoction: Menstrual cramps, Infusion/bath: uterus inflammation	Whole plant, leaf, flower	0.410	428, 827
ASCLEPIADACEAE				
Calotropis procera (Willd.) R. Br.<br>Algodão-brabo or algodão-de-seda	Decoction: Diarrhea, worm	Flower, fruit	0.535	430
ASTERACEAE				
Acanthospermum hispidum DC.<br>Espinho-de-cigano or federação or rapina	Syrup: Bronchitis, asthma, pneumonia, Decoction: numerous types of inflammations, cancer (not specified)	Leaf, flower, root	1.089	437, 441
Argyrovernonia harley (H. Rob.) Macheish.<br>Moricica	Decoction: Gastritis, ulcerous	Leaf, root	0.410	439, 447, 449
Bidens sp.	Decoction: Inflammation (Numerous types of inflammations)	Leaf, flower	0.267	431
Pluchea sp.<br>Mar-de-cravo	Decoction: High blood pressure, flu, kidneys infection	Leaf, flower, fruit	0.803	438
Verbesina chalybaea Mart. ex DC.<br>Balaio	Decoction: Dropsy, bladder infections	Leaf, root	0.535	442
Verbesina diversifolia DC.<br>Assa-peixe	Decoction: Kidneys stone	Fruit, seed	0.267	446
BEGONIACEAE				
Begonia reniformis Dryand.	Infusion: High blood pressure	Flower	0.267	451
BIGNONIACEAE				
Tabebuia aurea (Manso) Benth. & Hook.	Decoction: Uterus inflammation, worm	Inner bark, leaf	0.535	455

F. ex S. Moore<br>Craibeira				
Tabebuia avellanedae Lorentz ex Griseb.<br>Pau-d'arco-roxo	Decoction/bath: Inflammation (Numerous types of inflammations), Decoction: heart (Cardiac problems)	Inner bark, flower	0.535	454
BOMBACACEAE				
Chorisia glaziovii (O. Kuntze.)<br>Barriguda-de-espinho	Decoction: Heart (Cardiac problems), high blood pressure	Inner bark, flower	0.410	459
BORAGINACEAE				
Cordia globosa (Jacq.) Kunth.<br>Chumbinho, piçarra or moleque-duro	Infusion or Syrup: Flu, hemorrhage, Decoction/gargle: sore throat	Leaf, flower	0.803	460, 469
Cordia leucocephala Moric.<br>Piçarra or Moleque-duro	Infusion or Syrup: Sedative, hemorrhage, Decoction/gargle: sore throat	Inner bark, flower	0.803	462, 466
Cordia multispicata Cham.<br>Chumbinho	Infusion or Syrup: Bronchitis, worm, high blood pressure	Leaf, flower	0.803	464, 465
Cordia cf. *tricotoma* (Vell.) Anab. ex Stend<br>Pau-de-morro	Infusion or Syrup: Scar, high blood pressure	Inner bark, leaf, flower	0.535	470, 471, 472
Cordia sp.<br>Folha-larga	Infusion or Syrup: Hemorrhoids	Flower	0.267	468
Heliotropium angiospermum Murr.<br>Crista-de-galo	Infusion or Syrup: Heart (Cardiac problems), high blood pressure	Leaf, flower, root	0.410	461, 467
Tournefortia rubicunda Salzm.<br>Canudeiro	Infusion/bath: Mycosis, itching	Leaf	0.410	829
BROMELIACEAE				
Hohembergia sp.<br>Gravatá-de-folha-roxa	Decoction: High blood pressure, high cholesterol levels	Leaf, flower	0.535	480
Tillandsia loliacea Mart. ex Schult.<br>Barba-de-bode	Decoction: Uterus hemorrhage, ulcerous	Whole plant	0.535	473, 479
Tillandsia recurvata (L.) L.<br>Barba-de-bode-pequeno	Decoction: Bloodshed	Whole plant	0.267	475
Tillandsia streptocarpa Baker<br>Braba-de-bode-grande	Decoction: Heart (Cardiac problems)	Whole plant	0.267	474
CACTACEAE				

Arrojadoa rhodantha (Guerke) Br. et Rose Rabo-de-raposa	Decoction ou macerated: Heart (Cardiac problems), gastritis, spleen infection	Stem, root	0.660	482, 487
Cereus jamacaru DC. Mandacaru	Macearado: Liver, kidneys infection	Stem, fruit, root	0.535	486
Opuntia palmadora Br. et Rose Quipá	Decoction: Asthma, worms, inflammation (Numerous types of inflammations)	Stem	0.803	488
Pilosocereus gounellei (Weber) Byl. et Rowl. Xique-xique	Decoction: Dropsy, prostate inflammation (Numerous types of inflammations)	Stem, flower	0.535	485
Pilosocereus tuberculatus (Werdem.) Byl. et Rowl. Caxacubri	Decoction: Kidneys infection	Stem	0.267	481, 483
CAESALPINIACEAE				
Bauhinia cheilantha (Bong.) Stend. Mororó	Decoction: Diabetes, inflammation (Numerous types of inflammations) blood make fine, sedative, rheumatism	Inner bark, leaf, flower	1.339	493, 494, 498, 504, 507
Caesalpinia ferrea Mart. ex Tul. Pau-ferro	Decoction: Sore throat, bronchitis, bloodness, swell, scar	Inner bark, flower, fruit, seed	1.339	495, 499, 505, 510
Caesalpinia microphylla Mart. Catingueira-rasteira	Decoction or macerated:Sexual impotence, rheumatism	Inner bark, leaf	0.535	469, 513
Caesalpinia pulcherrima L. Sw. Maravilha	Decoction: Gastritis	Leaf, flower	0.267	503
Caesalpinia pyramidalis Tul. Catingueira	Decoction: Gases, bad digestion	Leaf, flower	0.410	491
Chamaecrista flexuosa (L.) Greene Capim-de-cobra	Decoction: Kidney infection	Root	0.267	501
Parkinsonia aculeata L. Turco	Decoction or Infusion: Flu, asthma, diabetes, high blood pressure	Inner bark, flower	1.071	500
Peltogyne pauciflora Benth. Pau-de-morro or jatobá	Decoction: Gases, flu, cough, sedative	Leaf, flower	1.071	463, 514
Senna obtusifolia (Vogel.) H. S. Irwin & Barneby Fedegoso	Decoction: Ulcerous	Leaf, root	0.267	509

Senna cf. *occidentalis* (L.) Link.<br>Mata-pasto	Decoction: Sore throat, bloodshed, gastritis, cancer	Leaf, root	1.071	492, 497
Senna splendida (Vogel.) H. S. Irwin & Barneby<br>Feijão-brabo or canafístula	Decoction: Kidneys infection, bronchitis, rheumatism, migraine, diarrhea, inflammation (Numerous types of inflammations), heart (Cardiac problems)	Inner bark, stem, leaf, flower, root	1.875	502, 512, 517, 830
Senna sp.1	Decoction: Liver	Leaf	0.267	515
Senna sp.2<br>Feijão-brabo	Decoction: Cough	Leaf, flower	0.267	516
CAPPARACEAE				
Capparis flexuosa (L.) L.<br>Feijão-brabo	Decoction: Flu, cough, pneumonia, rheumatism	Leaf, flower, fruit	0.928	529, 530, 531
Capparis jacobinae Moric.<br>Icó-verdadeiro or icó-preto	Decoction: Intoxication, fever, diabetes, diarrhea, pulmonary inflammation, bronchitis, heart (Cardiac problems)	Leaf, flower, fruit	1.589	520, 523, 524, 525, 526
Cleome diffusa Banks. ex DC.<br>Mussambê or mussambê-pequeno or mussambê-amarelo-pequeno	Decoction: Expel catarrh, flu, bronchitis, asthma, high blood pressure, cough	Whole plant, leaf, flower	1.178	518, 519, 522, 527, 528, 532
Cleome spinosa Jacq.<br>Mussambê-branco	Decoction or Infusion: Flu, bronchitis	Whole plant	0.535	521
CELASTRACEAE				
Maytenus rigida Mart.<br>Bom-nome	Decoction: Sexual impotence, rheumatism	Leaf, flower	0.535	533
COCHLOSPERMACEAE				
Cochlospermum sp.	Decoction: Pneumonia, uterus inflammation	Inner bark, leaf	0.535	534
COMBRETACEAE				
Combretum cf. *duarteanum* Cambess	Decoction: Expel catarrh	Leaf, flower	0.267	535
Combretum sp.	Decoction: Inflammation (Numerous types of inflammations), tumor	Stem, leaf	0.535	536
COMMELINACEAE				
Commelina cf. *erecta* L.<br>Erva-de-sta. Luzia	Decoction: Diabetes, rheumatism, eye inflammation, high blood pressure	Leaf, flower, root	1.071	537, 538, 530, 540

CONVOLVULACEAE				
Evolvulus sp.1<br>Cipó-marrapé or erva-branca	Decoction: Sedative, diarrhea, liver	Leaf, flower	0.660	542, 551
Jacquemontia sp.<br>Cipó-marrapé or erva-de-besta	Infusion: Kidneys infection, bronchitis	Leaf, flower, root	0.535	550, 552
Ipomoea sp.1<br>Algodão-preto	Decoction: Liver	Leaf, flower	0.267	545
Ipomoea sp.2<br>Cipó-cesto or batata-de-porco or jitirana or salsa	Decoction: High blood pressure, heart (Cardiac problems), sedative	Leaf, flower	0.660	544, 555, 557, 548
Ipomoea sp.3	Decoction: Sedative, high blood pressure, heart (Cardiac problems)	Leaf, flower	0.660	547, 549, 553
Operculina sp.<br>Batata-de-purga	Decoction: Intestinal cleansing, pain tooth	Root	0.535	546
CUCURBITACEAE				
Mormodica charantia L.<br>Melão-de-São Caetano	Decoction/bath: Venereal disease, louse, scabies	Leaf, flower	0.803	558, 561
DIOSCOREACEAE				
Dioscorea sincorensis R. Knuth<br>Salgueiro	Decoction: Kidneys infection, blodness, sore throat, diarrhea	Leaf, flower	1.071	563, 564
ERITHROXYLACEAE				
Erythroxylum revolutum Mart.<br>Araça-brabo	Decoction: Aphrodisiac, heart (Cardiac problems), high blood pressure	Leaf, flower	0.660	565
EUPHORBIACEAE				
Acalypha multicaulis Muell. Arg.<br>Canela-de-nambu	Decoction: Hemorrhage, pain tooth, inflammation (Numerous types of inflammations)	Inner bark	0.660	585, 594
Acalypha sp.<br>Canela-de-nambu	Decoction: Venereal disease	Root	0.267	600
Chamaesyce hyssopifolia Small<br>Porca-parideira	Decoction: Flu, cough, gastritis, heart (Cardiac problems), to expel placenta	Leaf, flower, root	1.339	572, 590, 593, 599
Cnidoscolus obtusifolius Pohl.<br>Favela or orelha-de-onça or faveleira-	Infusion: Cancer, liver, tumor, uterus inflammation	Leaf, flower	0.928	571, 582

mansa				
Croton argyrophylloides Müll. Arg.<br>Marmeleiro-branco	Decoction: Diabetes, Decoction/bath: inflammation (Numerous types of inflammations)	Inner bark	0.535	573, 597
Croton glandulosus L.	Decoction: Nasal bloodshed, sinusitis	Leaf, flower	0.392	592
Croton micans (Sw. em.) Muell. Arg.<br>Alecrim-de-vaqueiro	Infusion/bath: Hear, sedative, flu	Leaf, flower	0.803	579, 581
Croton rhamnifolius Humb., Bonplan & Kunth<br>Pau-de-leite or velame	Infusion: Flu, cough	Stem, leaf, flower	0.535	575, 576
Croton sonderianus Müll. Arg.<br>Marmeleiro-preto	Decoction: Stomachache, vomit (nausea), diarrhea	Bark	0.517	574
Euphorbia comosa Vell<br>Barbaça	Macerated: Bronchitis, menstrual cramps, inflammation (Numerous types of inflammations), cirrhosis, liver	Leaf, flower, root	1.196	568, 577, 587, 595
Euphorbia phosphorea Mart.<br>Pau-de-leite	Decoction: Blood make fine	Stem	0.267	584
Euphorbia sp.<br>Bredo-de-porco	Decoction: Cramps, intestine infection	Root	0.392	401
Jatropha mollissima (Pohl.) Baill.<br>Pinhão-de-seda or pinhão-branco or pinhão-brabo	Macerated: Kidneys infection, appetite	Leaf, flower, fruit	0.535	567, 570, 589
Jatropha mutabilis (Pohl.) Baill.<br>Pinhão-de-seda	Decoction: Intestinal cleansing, blood cleansing	Leaf, flower	0.535	580
Jatropha ribifolia (Pohl.) Baill.<br>Pinhão or pinhão-de-seda	Decoction: Diabetes, uterus inflammation	Leaf, flower	0.535	569, 601
Manihot glaziovii Muell. Arg.<br>Maniçoba	Macerated or Infusion: Headache, kidneys infection.	Leaf, flower	0.803	583, 591
Sebastiania sp.<br>Araça-brabo	Decoction: Diarrhea, kidneys infection, bronchitis	Stem, leaf	0.803	578, 598
Tragia cf. *bahiensis* Muell. Arg.<br>Tamarina	Decoction: Ulcerous	Whole plant	0.267	596
FABACEAE				

Andira sp.<br>Angelim-amargoso	Decoction: Blood make fine, high blood pressure	Inner bark	0.535	605, 677
Dioclea grandiflora Mart. ex Benth.<br>Mucunã	Fruto no umbigo: Umbilical hernia, Macerated/Bath: sedative, Macerated: flu, bronchitis	Leaf, flower, fruit	1.071	609, 615
Dioclea sp.<br>Mucunã	Infusion: Blood make thin, flu, bronchitis, heart (Cardiac problems)	Leaf, flower, fruit	1.071	604, 833
Erythrina velutina Willd.<br>Mulungu	Decoction: Headache, fever, sedative, milk maternal production	Inner bark, flower	0.928	619
Indigofera sp.<br>Anis-pequeno	Decoction: Kidneys stone, sedative	Leaf, flower	0.535	607, 608
Macroptilium lathyroides (L.) Urb.<br>Orelha-de-rato	Decoction: Hemorrhage	Leaf, flower	0.267	611
FLACOURTIACEAE				
Casearia sylvestris Swartz.	Decoction/bath:Woman hemorrhage	Inner bark, flower	0.267	621
LAMIACEAE				
Hyptis mutabilis Briq.<br>Sambacaita	Decoction/bath: Uterus inflammation	Leaf, flower	0.267	622
Leonotis nepetifolia (L.) R. Br.<br>Cravinho or cordão-de-frade	Decoction: Urinate keep	Leaf, flower	0.267	630
Ocimum basilicum L.<br>Manjericão-roxo	Decoction: Bronchitis, cough	Leaf, flower	0.410	623
Ocimum gratissimum L.<br>Alfavaca or alfavaca-branco	Infusion: Bad digestion, flu, cough	Leaf, flower	0.928	624, 629
Ocimum tenuiflorum L.<br>Alfavaca	Decoction or Infusion: Cholesterol, high blood pressure	Leaf, flower	0.535	626
Raphiodon echinus (Nees & Mart.) Schauer.<br>Flor-de-urubu	Decoction/bath: Uterus inflammation	Leaf, root	0.267	625
LILIACEAE				
Smilax sp.<br>Cipó-de-japecanga	Macerated: Scar	Flower, fruit	0.267	632
LOASACEAE				

Loasa rupestris Gardner<br>Urtiga-de-mocó	Decoction/bath: to expel placenta	Leaf, flower	0.267	633
MALPIGHIACEAE				
Byrsonima gardneriana Juss.<br>Murici	Decoction: Blood make fine, flu, sore throat	Leaf, flower, fruit	0.660	636, 637
Byrsonima cf. *intermedia* Juss.<br>Pitombinha or cipó-de-rego	Decoction: Gallstone, Kidneys stone, prostate inflammation, bad digestion, worm	Leaf, flower	1.053	638, 640, 647
Byrsonima sp.<br>Murici	Decoction: Flu, sore throat	Leaf, flower	0.410	643
Galphimia sp.	Decoction: Sore throat, bronchitis, hoarseness, urinate keep, worm	Leaf, flower, fruit	1.196	639, 642, 645
MALVACEAE				
Gaya aurea St. -Hill.	Infusion: Bad digestion	Leaf, flower	0.267	654
Herissantia tiubae (K. Schum.) Briz.<br>Lava-prato	Infusion: Flu, fever	Leaf, flower	0.535	649
Sida cordifolia L.<br>Malva-amarela or mela-bode	Decoction: Kidneys infection	Root	0.267	650
Sida sp.1<br>Barba-de-boi	Infusion: Bad digestion, gases	Leaf, flower	0.410	652
Sida sp.2<br>Malva-branca	Decoction/bath: Scar	Leaf, flower	0.267	651
MARCGRAVIACEAE				
Norantia brasiliensis Choisy	Decoction: Heart (Cardiac problems)	Leaf, flower	0.267	658
MELIACEAE				
Melia azedarach L.<br>Lírio	Syrup: Flu, high blood pressure	Flower	0.535	660, 834
MIMOSACEAE				
Acacia bahiensis Benth.<br>Carcará or angico-monjolo	Infusion: Uterus infection, blood make fine, rheumatism, epidermis, venereal disease	Inner bark, flower	1.339	662, 665, 678
Acacia sp.<br>Espinheiro-branco	Decoction/bath: Uterus infection	Inner bark	0.267	671
Anadenanthera colubrina (Vell.) Brenan	Decoction or Syrup: Bronchitis, pulmonary	Inner bark	0.660	672, 675

Angico-de-caroço	inflammation, flu			
Chloroleucon sp. Arapiraca	Decoction: Heart (Cardiac problems)	Inner bark, flower	0.267	666, 676
Mimosa caesalpinifolia Benth. Cascudo	Decoction or macerated: Inflammation (Numerous types of inflammations), high blood pressure	Inner bark, flower	0.535	669
Mimosa tenuiflora (Willd.) Poir. Jurema-preta	Decoction: Inflammation (Numerous types of inflammations), fever, menstrual cramps	Inner bark, flower	0.660	670
Mimosa sp. Jurema-branca	Decoction or Syrup: Asthma, flu	Inner bark, flower	0.535	680
Paraspiptadenia zehntneri (Harms) M. P. Lima & H. C. Lima Angico-monjola	Decoction/bath: Flu, uterus inflammation	Inner bark	0.535	667
Piptadenia sp.1 Arranhento	Decoction: Sedative, high blood pressure	Inner bark, leaf, flower	0.534	668, 674
Piptadenia sp.2 Angelim-verdadeiro	Decoction: Gastritis	Inner bark	0.267	664
Pithecellobium diversifolium Benth. Corcarozeiro	Decoction: Diabetes, bronchitis, Decoction/bath: uterus inflammation	Inner bark	0.803	661, 673, 681
MYRTACEAE				
Eugenia citrifolia Poiret. Araça-verdadeiro	Decoction: Migraine, heart (Cardiac problems)	Leaf	0.535	682
NYCTAGINACEAE				
Boerhavia diffusa L. Pega-pinto	Decoction/bath: Genital secretion, genital infection	Root	0.535	683
OLACACEAE				
Ximenia americana L. Ameixa	Decoction or tincture: Inflammation (Numerous types of inflammations), bloodshed, backache	Inner bark	0.803	684
ONAGRACEAE				
Ludiwigia sp.	Decoction: Liver, stomachache	Flower. fruit	0.410	685
OXALIDACEAE				

Oxalis sp.1<br>Umbuzeirinho	Decoction: Asthma, flu, cough, intestinal cleansing	Leaf, flower, root	0.958	687, 688, 689, 692
Oxalis sp.2<br>Azedinho	Decoction: Expel catarrh, prostate inflammation	Leaf, flower, root	0.535	690, 692
PAPAVERACEAE				
Argemone mexicana L.<br>Cardinho or cardo-santo	Decoction: Pneumonia, uterus inflammation	Whole plant, leaf, root	0.535	693, 694
PASSIFLORACEAE				
Passiflora foetida L.<br>Maracujá-papoco	Decoction or Infusion: Heart (Cardiac problems), sedative	Leaf, flower	0.535	696, 697, 698
Passiflora sp.<br>Margarida	Decoction: Sedative, high blood pressure	Leaf, flower	0.535	695
PHYTOLACACEAE				
Petiveria sp.<br>Tipi	Infusion: Sedative, heart (Cardiac problems)	Leaf, flower	0.535	700
Microtea sp.<br>Angélica-rasteira	Decoction: Sedative, to expel placenta	Leaf, flower, root	0.535	699, 701, 702
PLUMBAGINACEAE				
Plumbago sp.	Decoction: Cramps, sedative, urinary infection, stomachache	Leaf, flower, root	0.928	704, 705, 706
POLYGALACEAE				
Polygala sp.<br>Pé-de-urubu or gelol or mudubinha	Decoction: Stoke, dismenorrhoea	Whole plant	0.535	711, 712
PONTEDERIACEAE				
Eichhornia paniculata Kunth.	Decoction: Hemorrhoids	Leaf	0.267	713
PORTULACACEAE				
Portulaca sp.1<br>Beldroega	Decoction: Kidneys infection	Root	0.267	719
Portulaca sp.2<br>Beldroega or bico-de-urubu	Decoction: Kidneys stone, gallstone	Root	0.535	715, 716, 717
Portulaca sp.3<br>Beldroega	Decoction: Eye inflammation, kidneys infection	Flower, root	0.535	721

Portulaca sp.4<br>Beldroega	Decoction: Kidneys infection	Leaf, flower, root	0.267	714, 722
Talinum paniculatum Gaertn.<br>Bredo	Decoction: Prostate inflammation	Whole plant	0.267	720
Talinum sp.<br>Bredo	Decoction: Kidney infection	Root	0.267	718
PUNICACEAE				
Punica granatum L.<br>Romã	Decoction/gargle: Sore throat	Fruit	0.267	723
RHAMNACEAE				
Gouania latifolia Reiss	Decoction: Uterus inflammation, backache	Inner bark, leaf	0.535	727
Ziziphus juazeiro Mart.<br>Juazeiro	Decoction: Flu, stomachache, bad digestion, Syrup: pneumonia, tuberculosis, bronchitis	Inner bark, bark, fruit	1.178	724, 725, 726, 728
RUBIACEAE				
Emmeorhiza umbelata (Spreng) K. Schu.	Decoction: Intoxication	Flower, fruit	0.267	746
Guettarda sp.	Decoction: Stomachache, inflammation (Numerous types of inflammations)	Leaf, flower	0.535	742
Tocoyena formosa (Cham. & Schltdl.) K. Schum.<br>Jenipaparana	Infusion: Liver, urinary infection, rheumatism, heart (Cardiac problems)	Inner bark, leaf, flower, fruit	1.071	734, 736, 739
Richardia sp.<br>Erva-de-besta	Decoction: Intestinal cleansing, sore throat, bronchitis, nasal bloodshed, rheumatism	Flower, root	1.195	729, 731, 733, 748
SAPINDACEAE				
Allophylus quercifolius (Mart.) Rasdlk.	Decoction: Ulcerous	Leaf, flower	0.267	756
Cardiospermum coridum L.<br>Timbó	Decoction: Ulcerous	Leaf, flower, fruit	0.267	757, 758
Cardiospermum oliveirae Ferruci.<br>Cipó-cruapé or timbó	Decoction: Ulcerous, to expel placenta, inflammation (Numerous types of inflammations), tumor	Stem, leaf, flower	1.071	755, 759, 762
Serjania sp.1<br>Timbó	Decoction: Ovary inflammation, scar, ulcerous, cramps	Leaf, flower	1.071	752, 754, 760, 763
Serjania sp.2	Decoction: Cancer (not specified)	Leaf, flower	0.267	761

Salgueiro				
SAPOTACEAE				
Sideroxylum obtusifolium (Roem. & Schult.)Penn.<br>Quixabeira	Decoction: Gastritis, stroke, inflammation (Numerous types of inflammations), Decoction/bath: genital inflammation	Inner bark	1.071	764, 765
SCROPHULARIACEAE				
Angelonia sp.1	Decoction: Kidneys infection	Root	0.267	768
Angeloina sp.2	Decoction: Inflammation (Numerous types of inflammations)	Leaf, flower	0.267	767
Angelonia sp.3	Decoction: high Cholesterol levels	Leaf, flower	0.267	769
SIMARUBACEAE				
Simaba mayana Casar<br>Pratudo	Decoction: Inflammation (Numerous types of inflammations), stroke	Leaf	0.535	770
SOLANACEAE				
Nicotiana glauca L.	Decoction: Bad digestion, Decoction/bath: uterus inflammation	Leaf, flower	0.535	774, 776
Solanum americanum L.	Decoction: Sore throat	Leaf, flower	0.267	771
Solanum sp.1<br>Jurubeba	Decoction: Kidneys inflammation	Stem, leaf, flower	0.267	775
Solanum sp.2<br>Tomatinho-do-campo	Decoction: Kidneys inflammation	Leaf, flower, fruit	0.267	772
STERCULIACEAE				
Byttneria sp.	Decoction: Flu, asthma, diabetes, high blood pressure	Inner bark, flower	1.071	751
Melochia tomentosa L.<br>Malva-vermelha	Infusion: Flu, cold, bronchitis, pulmonary inflammation	Leaf, flower	0.785	777, 779, 780, 782
Melochia sp.<br>Malva-branca	Infusion: Flu, liver	Leaf, flower	0.535	778
Walteria sp.<br>Malva-branca	Infusion: Scar	Leaf, flower	0.267	781
TURNERACEAE				
Turnera sp.	Decoction: Intestinal cleansing, bronchitis	Leaf, flower	0.535	783, 785

Flor-de-catenga or chanana				
UMBELIFERAE				
Anethum graveolens L.<br>Endro	Infusion: Diarrhea	Flower	0.267	789
URTICACEAE				
Dendrocnide sp.<br>Urtiga	Decoction: Ulcerous	Whole plant	0.267	791
VERBENACEAE				
Lantana sp.<br>Camará or chumbinho or moleque-duro	Decoction: Flu, fever, asthma, cough, bronchitis, diabetes, high blood pressure, sedative	Leaf, flower	1.857	792, 793, 798, 804
Lippia sp.<br>Alecrim-de-vaqueiro or alecrim-de-caboclo or alecrim-pimenta or chumbinho	Decoction: Flu, heart (Cardiac problems), Syrup: bronchitis, expel catarrh, pulmonary inflammation, pneumonia, Decoction/gargle: sore throat	Leaf, flower	1.428	794, 796, 800, 801, 803, 805, 806
Stachytarfeta sp.	Decoction: Urinate keep, kidneys infection	Whole plant	0.535	797
VITACEAE				
Cissus bahiensis Lombardi	Decoction: Sexual impotence, rheumatism	Leaf, flower	0.535	815
Cissus decidua Lombardi<br>Embiratanha	Decoction: Cancer (not specified), uterus inflammation	Stem, leaf, flower	0.535	814
Cissus erosa L. C. Rich.<br>Cipó-parreiro	Decoction: Liver, Decoction/bath: menstrual cramps	Leaf, flower	0.535	810
Cissus simsiana Schult. & Schult. F.<br>Cipó-parreira	Decoction: Intestinal cleansing, high blood pressure	Leaf, flower	0.660	808, 812, 813
Cissus verticillata subsp. *verticillata* (L.) Nicolson & C. E. Jarvis<br>Insulina	Decoction: Cirrhosis, liver, diabetes, cholesterol	Leaf, flower, fruit	0.928	809, 811
ZIGOPHILACEAE				
Kallstroemia tribuloides Wight & Arn.	Decoction: Heart (Cardiac problems)	Root	0.267	816

ZINGIBERACEAE				
Alpinia speciosa Schum.<br>Colônia	Infusion: Migraine, heart (Cardiac problems)	Leaf	0.535	817
